# Neighborhood disadvantage and 30-day readmission risk following *Clostridioides difficile* infection hospitalization

**DOI:** 10.1186/s12879-020-05481-x

**Published:** 2020-10-16

**Authors:** Elizabeth Scaria, W. Ryan Powell, Jen Birstler, Oguzhan Alagoz, Daniel Shirley, Amy J. H. Kind, Nasia Safdar

**Affiliations:** 1 Department of Industrial and Systems Engineering, University of Wisconsin-Madison, 1513 University Avenue, Madison, WI 53706 USA; 2 Division of Geriatrics and Gerontology, Department of Medicine, University of Wisconsin-Madison, School of Medicine and Public Health, 1685 Highland Avenue, 5158 Medical Foundation Centennial Building, Madison, WI 53705 USA; 3 Department of Biostatics & Medical Informatics, University of Wisconsin-Madison, School of Medicine and Public Health, 201 WARF Building, 610 Walnut Street, Madison, WI 53726 USA; 4 Department of Population Health Sciences, University of Wisconsin-Madison, School of Medicine and Public Health, 707 WARF Building, 610 Walnut Street, Madison, WI 53726 USA; 5 Division of Infectious Diseases, Department of Medicine, University of Wisconsin-Madison, School of Medicine and Public Health, 1685 Highland Avenue, 5158 Medical Foundation Centennial Building, Madison, WI 53705 USA; 6 Geriatric Research Education and Clinical Center, William S. Middleton Memorial Veterans Hospital, 2500 Overlook Terrace, Madison, WI 53705 USA; 7 William S. Middleton Memorial Veterans Hospital, 2500 Overlook Terrace, Madison, WI 53705 USA

**Keywords:** *Clostridioides difficile*, Socioeconomic disadvantage, Social determinants of health, Medicare

## Abstract

**Background:**

*Clostridioides difficile* infection (CDI) is commonly associated with outcomes like recurrence and readmission. The effect of social determinants of health, such as ‘neighborhood’ socioeconomic disadvantage, on a CDI patient’s health outcomes is unclear. Living in a disadvantaged neighborhood could interfere with a CDI patient’s ability to follow post-discharge care recommendations and the success probability of these recommendations, thereby increasing risk of readmission. We hypothesized that neighborhood disadvantage was associated with 30-day readmission risk in Medicare patients with CDI.

**Methods:**

In this retrospective cohort study, odds of 30-day readmission for CDI patients are evaluated controlling for patient sociodemographics, comorbidities, and hospital and stay-level variables. The cohort was created from a random 20% national sample of Medicare patients during the first 11 months of 2014.

**Results:**

From the cohort of 19,490 patients (39% male; 80% white; 83% 65 years or older), 22% were readmitted within 30 days of an index stay. Unadjusted analyses showed that patients from the most disadvantaged neighborhoods were readmitted at a higher rate than those from less disadvantaged neighborhoods (26% vs. 21% rate: unadjusted OR = 1.32 [1.20, 1.45]). This relationship held in adjusted analyses, in which residence in the most disadvantaged neighborhoods was associated with 16% increased odds of readmission (adjusted OR = 1.16 [1.04, 1.28]).

**Conclusions:**

Residence in disadvantaged neighborhoods poses a significantly increased risk of readmission in CDI patients. Further research should focus on in-depth assessments of this population to better understand the mechanisms underlying these risks and if these findings apply to other infectious diseases.

## Introduction

*Clostridioides difficile* is a major cause of healthcare-associated diarrhea in the United States, responsible for nearly 500,000 cases of *C. difficile* infection (CDI), 30,000 deaths and over $5 billion each year [1, 2]. One reason that curtailment of CDI remains a major challenge is the high rate of CDI recurrence, which occurs in up to 30% of patients [3]. Patients with recurrent CDI may need to be subsequently readmitted to a healthcare facility, which presents an opportunity for continued transmission of CDI in the inpatient setting and new infections in susceptible hosts. Data on readmissions following an inpatient stay with CDI, while limited, show that approximately 23% of patients had at least one readmission, with approximately 32% of readmissions carrying a principal diagnosis of CDI [4]. Patients with a CDI discharge have been found to have a 16 percentage point higher rate of 30-day readmission than patients without a CDI discharge [5].

Commonly cited risk factors of recurrent CDI include older age and continued use of antibiotics [6]. Few studies that focus on risk factors of CDI recurrence consider the impact of social determinants of health. Social determinants are increasingly recognized as major contributors to readmissions for chronic conditions like congestive heart failure and myocardial infarction. However, little data exist on the relationship between social determinants of health and outcomes of acute infectious conditions (other than pneumonia-a condition that Centers for Medicare & Medicaid Services (CMS) penalizes for readmission) in developed countries such as the US. Because CDI diagnosis is associated with high rates of recurrence, it is plausible that the rate of readmission following a CDI-related stay would be similar to that for chronic conditions and that socioeconomic disadvantage would be an important contributor to the risk of recurrence and thereby readmission.

Socioeconomic disadvantage likely adversely impacts a CDI patient’s post discharge course. Challenges may include financial constraints to completing the full antibiotic course, especially as the most common treatments for CDI, oral vancomycin and fidaxomicin, can be prohibitively expensive for uninsured patients [7, 8]. Other challenges may include inability to manage environmental cleaning to reduce re-infection and spore shedding, and lack of resources such as transportation and social support to facilitate follow-up care [7, 9]. Household crowding, a common indicator of socioeconomic disadvantage, has also been linked to poorer outcomes for infectious disease patients [10]. As CDI is a disease caused by perturbation of the gut microbiome, factors that impede restoration of the gut microbiome may also affect CDI outcomes. Patients living in socioeconomically disadvantaged neighborhoods have higher rates of comorbid conditions, increasing their contact with the healthcare system and risk of CDI and/or antibiotic exposure. A growing body of literature suggests that diet is a major driver of gut microbiome composition and health [11, 12]. Patients living in socioeconomically disadvantaged neighborhoods are more likely to live in ‘food deserts,’ where access to healthy, high fiber foods may be limited [13]. Consumption of low-fiber, highly processed foods has been found to be strongly linked to socioeconomic disadvantage [14]. As these potential mechanisms impact specific characteristics of CDI, it is likely that disadvantage would adversely affect a CDI patient’s outcomes. Therefore, we hypothesized that socioeconomic disadvantage as measured by residence in a disadvantaged neighborhood would be associated with a higher risk of readmission for CDI patients.

## Methods

We conducted a retrospective observational study to evaluate the association between neighborhood socioeconomic disadvantage and risk of readmission for patients discharged after a CDI-related stay.

### Data source and cohort creation

We measured the relative socioeconomic disadvantage at the Census Block Group, or ‘neighborhood,’ level using the Area Deprivation Index (ADI). ADI was developed three decades ago and subsequently has been validated at the more granular Census Block Group level [14]. ADI is a composite index that draws from several weighted indicators of neighborhood socioeconomic disadvantage, including constructs of income, employment, education and housing quality [15]. ADI is validated at the neighborhood level and has been used in several other studies [16–18].

**Fig. 1:**
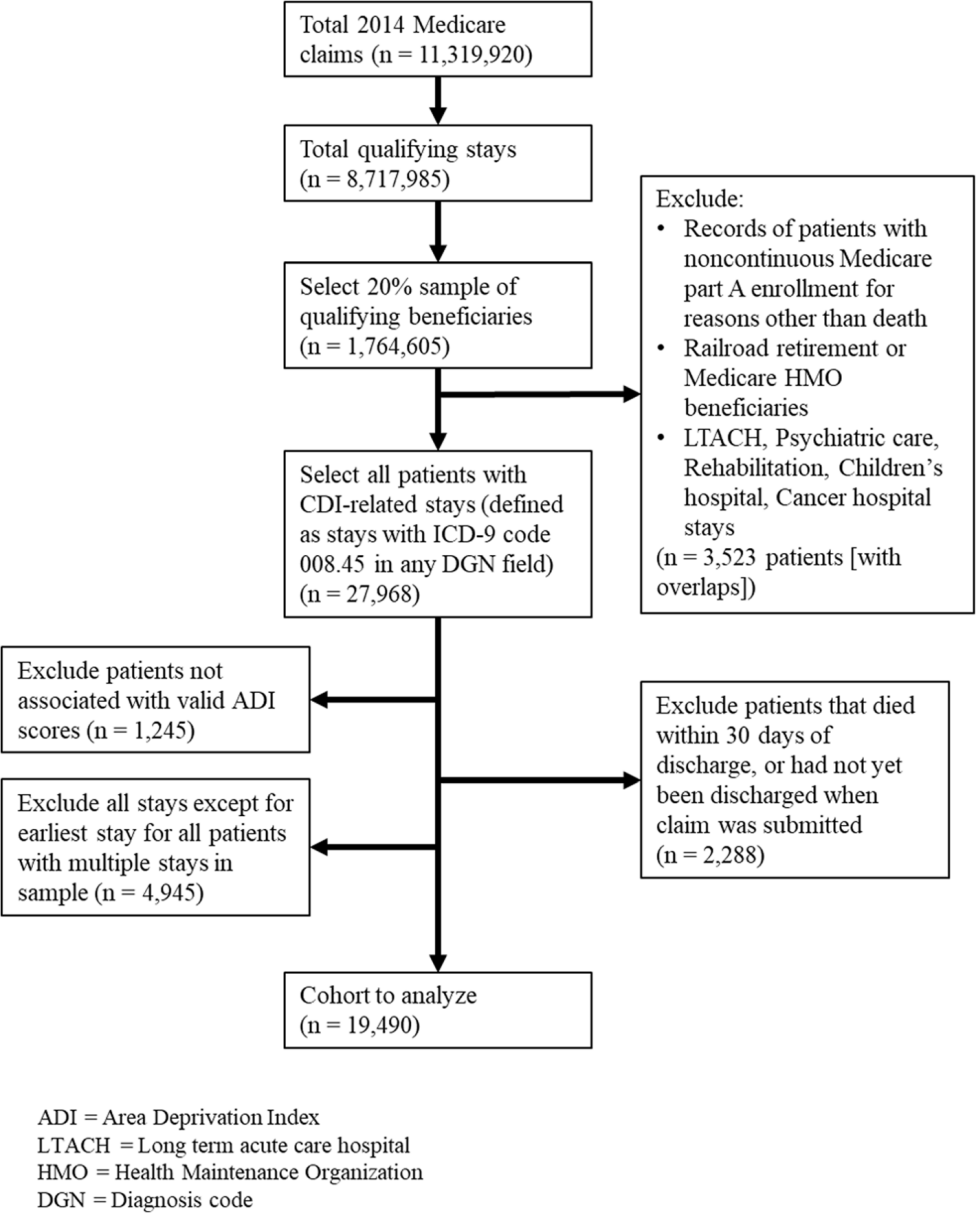
Creation of the analyzed cohort and application of exclusion criteria

The cohort included all patients within a 20% Medicare claims national random sample who had a CDI-related index inpatient stay (ICD-9-CM = 008.45) between January 1–November 30, 2014. Beneficiaries with valid ZIP+ 4 codes (95%) were geolinked to corresponding neighborhood national ADI ranking, obtained from the UW Neighborhood Atlas [19]. As consistent with CMS readmission metric policy, patients not meeting the following criteria were excluded: stays from non-federal short-term acute hospitals, those without continuous Medicare part A and B enrollment, those who died during their index stay, or were discharged against medical advice. Other exclusions included: patients without valid geolinked ADI national percentile scores, age less than 18 years, those with railroad retirement benefits, or those enrolled in Medicare HMO. As multiple hospital stays by a single patient could invalidate the assumption of independence for all records in our dataset, we retained only the first stay for patients that had multiple hospital stays during the study period. Figure 1 describes the creation of our study cohort.

### Variables

The primary outcome measure was odds for 30-day all-cause readmission as defined previously and which is used by CMS to inform readmission-based policies [20, 21]. We considered all-cause readmissions because of the wide range of reasons patients with recurrent CDI can be readmitted. Our key explanatory variable was neighborhood-level socioeconomic disadvantage as measured by ADI.

Covariates selected for model adjustment were chosen based on previous literature and conceptual models of readmission [22, 23]. Illness burden was captured via Elixhauser comorbidity categories in the 12 months prior to each index hospitalization [24]. Patients were considered dual Medicare-Medicaid enrolled if they were enrolled in Medicaid in any of the 12 months preceding index hospitalization. Patients were considered disabled if their reason for Medicare entitlements was disability related. Additional patient-level adjustments were made for age, race/ethnicity, and rurality via Rural-Urban Commuting Area (RUCA) codes. Age was split at 65 as advanced age is widely cited as a risk factor for incident and recurrent CDI [3, 25]. Factors associated with hospital stay included length of stay (LoS) and discharge to a skilled nursing facility (SNF) after index hospitalization. Factors related to hospital characteristics included hospital Medicare beneficiary discharge volume (grouped into tertiles), medical school affiliation (major, minor, or none), and hospital type (non-profit, for-profit, government).

### Statistical analyses

We graphically depicted the 30-day readmission rate as a function of patient ADI national ranking to evaluate the unadjusted relationship between the outcome and our key explanatory variable. The cohort was split into two groupings on the basis of neighborhood disadvantage; patients living in the 85% least disadvantaged neighborhoods and those living in the 15% most disadvantaged neighborhoods. The threshold of 85 was selected based on existing literature that showed patients living in the most disadvantaged 15% of neighborhoods had significantly increased risk of readmission for common chronic conditions [22]. To test the stability of results, we conducted a sensitivity analysis on the choice of threshold.

For both ADI groupings of neighborhood disadvantage, we examined descriptive means and proportions to assess how the two groups differ on key baseline patient and hospital characteristics. We then used multivariable logistic regression techniques to further examine the relationship between ADI grouping and 30-day readmission rate.

All odds ratios and predicted probabilities were calculated twice; once using a generalized estimating equations approach with clustered standard errors at the hospital-level and once using a general linear model with robust standard errors [26, 27]. Since no difference was found between the results of the two models, we present the general linear model results. We calculated predicted probabilities using marginal standardization methods [28].

Finally, we performed a series of subgroup analyses on a-priori subgroups that may have a differential odds of readmission for those living in the most disadvantaged neighborhoods using established methods to test for interaction effects [29]. These groups were patient dual Medicare-Medicaid enrollment status, SNF discharge status and race. Race and dual Medicare-Medicaid enrollment were analyzed because of their association with increased likelihood of residence in a disadvantage neighborhood as previously reported [22]. Discharge to SNF status was analyzed to assess the impact of the patient’s post-discharge environment, as patients living in disadvantaged neighborhoods that are discharged to SNFs could be insulated from any adverse effects of their residential environment. Analyses were conducted using SAS version 9.4 (SAS Institute) and StataSE 15 (StataCorp).

### IRB approval

This study has been approved by the University of Wisconsin Health Sciences Institutional Review Board.

## Results

### Cohort creation and characteristics

**Fig. 2:**
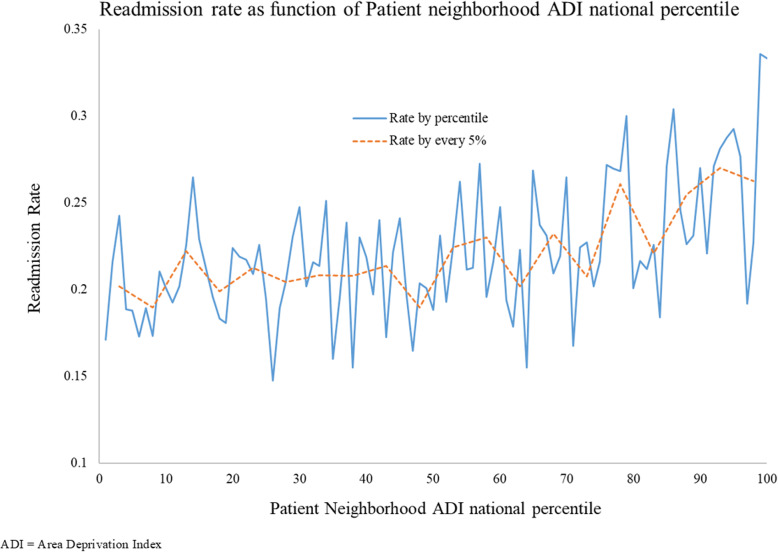
Unadjusted readmission rate as a function of patient neighborhood ADI. The dotted line shows the unadjusted readmission rate over ever five percentiles

Our final cohort included 19,490 unique Medicare-insured patients with a CDI-related index stay discharged from 2855 unique health care facilities (see Fig. 1 for detailed sample derivation). Less than 0.04% of patients in the cohort were missing age and LoS data. Patients with missing age or LoS data were grouped into the 65 years and above age category or the fewer than 3 days LoS category, respectively. From our cohort, 22% were readmitted within 30 days (unadjusted). Patients living in the most disadvantaged 15% of neighborhoods were readmitted at an observed rate of 26%, while patients living in the least disadvantaged 85% were readmitted at an observed rate of 21%. Figure 2 depicts the observed readmission rate over the range of ADI national percentiles.

Descriptively, the most disadvantaged 15% of neighborhoods had higher rates of dual Medicare-Medicaid enrollment, of patients younger than 65 years, and of patients of black race relative to the least disadvantaged 85% of neighborhoods (Table 1). This patient population also had higher rates of nearly all comorbidities, including chronic conditions such as diabetes and hypertension.

**Table 1:** Characteristics of Medicare patients with a CDI-related stay by ADI national percentile

Variable	ADI National percentile < 85 (***n*** = 17,094)	ADI National percentile ≥ 85 (***n*** = 2396)
**Patients**		
Age		
Mean Age at Discharge (SD), y	75.64 (12.83)	71.79 (13.90)
18–65 y	16%	26%
65+ y	84%	74%
Sex		
Male	39%	39%
Female	61%	61%
Race		
White	83%	58%
Black	10%	28%
Other/Unknown	8%	14%
Medicaid Enrollment		
Not Medicaid Enrolled	74%	51%
Medicaid Enrolled	26%	49%
Disability		
Not disabled	72%	57%
Disabled	28%	43%
Patient RUCA		
Urban core	73%	65%
Suburban	9%	6%
Large rural	10%	14%
Small rural	8%	15%
Elixhauser Comorbidities		
Hypertension	79%	84%
Fluid and electrolyte disorders	59%	65%
Deficiency anemia	52%	58%
Diabetes (without chronic complications)	37%	46%
Renal failure	36%	42%
Chronic pulmonary disease	34%	42%
Congestive heart failure	30%	36%
Depression	26%	29%
Other neurological conditions	25%	28%
Hypothyroidism	25%	23%
Peripheral vascular disease	22%	26%
Weight loss	20%	25%
Obesity	17%	21%
Diabetes (with chronic complications)	17%	24%
Valvular disease	15%	14%
Metastatic cancer	5%	4%
Alcohol abuse	4%	6%
Drug abuse	4%	6%
Chronic blood loss anemia	4%	5%
Lymphoma	3%	3%
Acquired immune deficiency syndrome	1%	2%
Pulmonary circulation disease	10%	11%
Rheumatoid arthritis/collagen vascular disease	8%	9%
Paralysis	8%	10%
Liver disease	7%	9%
Solid tumor without metastasis	14%	11%
Psychoses	11%	14%
Coagulopathy	14%	15%
**Index Stay**		
Length of Stay		
Mean Hospital length of stay (SD), days	9.68 (9.60)	10.38 (11.80)
≤ 2 days	8%	7%
3–4 days	21%	18%
5–6 days	18%	18%
7+ days	53%	56%
SNF Discharge		
Discharged to SNF	37%	33%
Not discharged to SNF	63%	67%
**Index Hospital**		
Medical School Affiliation		
Hospital affiliated with medical school	50%	56%
Minor medical school affiliated	27%	30%
Major medical school affiliated	23%	26%
Hospital Type		
Non-profit hospital	75%	68%
For profit hospital	13%	17%
Government hospital	12%	15%
Discharge		
Hospital discharge volume in 2014 (SD)	6463.18 (4791.26)	6554.34 (4968.94)
Hospital discharge volume: lowest tertile	8%	10%
Hospital discharge volume: middle tertile	23%	21%
Hospital discharge volume: highest tertile	69%	69%
**Outcome**		
Rate of 30-day readmission	21%	26%
Rate of 30-day death	13%	13%

*ADI* Area Deprivation Index

*LOS* Length of Stay

*RUCA* Rural -Urban Commuting Area

*SNF* skilled nursing facility

Patients living in the 15% most disadvantaged neighborhoods also had higher rates of discharge from a hospital with relatively low discharge volumes. Patients living in the 85% least disadvantaged neighborhoods had a higher rate of discharge to a SNF relative to patients living in the most disadvantaged 15% of neighborhoods. These patients also had a higher rate of residence in an urban neighborhood.

### Patient neighborhood disadvantage and 30-day readmission risk

Patients living in the most disadvantaged neighborhoods had a significantly increased odds of 30-day readmission compared to patients living in less disadvantaged neighborhoods (unadjusted OR = 1.32, 95% CI: [1.20, 1.45]) (Table 2). When adjusted for all covariates, living in the most disadvantaged neighborhoods was associated with a 16% increased odds of readmission relative to those from the least disadvantaged neighborhoods (OR = 1.16, 95% CI: [1.04, 1.28]). This translates into a 2.5% increase in the predicted probability of a readmission (from 21.6 to 24.1%). The effect is similar in magnitude to that of diabetes with chronic complications (OR = 1.12, 95% CI [1.01, 1.25]) and renal failure (OR = 1.19, 95% CI: [1.10, 1.29]) (see Supplementary Table 1). Results were robust to the choice of threshold: patients living in the least advantaged neighborhoods were consistently estimated to be at greater risk (Supplementary Table 2).

**Table 2:** Odds of 30-day readmission for CDI patients by ADI score national percentile

Variable	Odds ratio (95% CI)	Predicted Probability (%) (95% CI)
Unadjusted		
ADI < 85 percentile	Reference	21.3 (20.7, 21.9)
ADI ≥ 85 percentile	1.32 (1.20, 1.45)	26.3 (24.5, 28.1)
Adjusted		
ADI < 85 percentile	Reference	21.6 (21.0, 22.2)
ADI ≥ 85 percentile	1.16 (1.04, 1.28)	24.1 (22.4, 25.8)

*ADI* Area Deprivation Index

Results of the subgroup analyses showed no evidence for a significantly modified effect by the beneficiary’s SNF discharge status, dual Medicare-Medicaid enrollment status, or race (Supplementary Table 3).

## Discussion

We found that living in a disadvantaged neighborhood was associated with increased odds of readmission for patients with a CDI-related index hospital stay and this remained true after adjustment for patient-, stay-, and hospital-level variables. To our knowledge, this is the first study to explore the relationship between social determinants of health as measured by neighborhood disadvantage and risk of readmission for patients with an index CDI-related hospitalization.

Our analyses found that both neighborhood disadvantage and dual Medicare-Medicaid enrollment (a proxy for low-income individuals and common indicator of social risk) were significant predictors of readmission. This suggests ADI captures a dimension of socioeconomic disadvantage that dual Medicare-Medicaid enrollment status and potentially other individual social risk factors cannot. This ability may be driven in part by the impact of neighborhood disadvantage on a patient’s ability to follow post-discharge care and the success probability of that care.

Our findings have implications for clinicians, infection preventionists, and healthcare institutions. For clinicians, the approaches to mitigating risk to CDI patients living in disadvantaged neighborhoods may need to vary from those applied to similar patients with chronic conditions. For example, follow-ups with primary care, potentially using telemedicine resources, might need to be conducted sooner compared to other conditions. Such approaches may need to be conducted in addition to those targeting the impact of neighborhood disadvantage across all conditions. From the infection prevention perspective, placing CDI patients in contact precautions and promoting enhanced hand hygiene practices by healthcare workers is variably effective in reducing transmission, in part because of challenges in high fidelity implementation and breaches in prevention practices. Therefore, preventing readmissions as an upstream intervention is key. Additionally, unlike many chronic conditions, readmission of patients with a contagious disease such as CDI has implications not just for individual patients but for all hospitalized patients including others at risk for readmission. Asymptomatic colonized patients have been shown to contribute significantly to the overall burden of CDI in healthcare institutions, emphasizing the need to prevent unnecessary readmissions [30]. Preventing readmissions for all patients is also important as a marker for quality and because of the financial implications related to increased rates of readmission. The rates of readmission in this study of Medicare enrollees with an index stay of CDI exceeds the rate of readmission of 17% found in the general Medicare population [31]. We also found that the length of stay in our study was approximately 3 days longer than that found in the general Medicare population [32]. As the US healthcare system moves to value-based purchasing with a reduced likelihood that payers will cover costs of readmissions, and the financial penalties to healthcare institutions for CDI, it is important to understand factors that increase readmission risk. The ability to identify specific patients with increased risk for readmission could be a valuable tool to allocate resources such as transitional care programs, intensive case management, and social work to those patients.

Our findings are supported by other studies of readmissions in CDI that report rates of 25–30% readmission and prolonged duration of hospitalization. In a retrospective cohort study of 385,682 initial CDI hospitalizations identified between years 2009 and 2013 in the 4 states included in the State Inpatient Database (AHRQ), 25.7% of patients required readmission; among these, 36.8% had recurrent CDI as the principal diagnosis at the time of readmission [33]. A study of data from the Healthcare Cost and Utilization Project (HCUP) saw that patients with a primary or secondary diagnosis of CDI had a 30-day readmission rate of 29.1% [34].

We were not able to determine the extent to which recurrent CDI was the cause of the readmissions. Given the high recurrence rate associated with CDI, it is plausible that recurrent CDI contributed to the readmissions for at least some patients. Prediction models for recurrent CDI have been developed but have had variable performance to consistently predict patients at risk for CDI [35–38]. These models have largely focused on patient level factors such as severity of CDI or comorbidities that may increase readmission risk. Our study examining the relationship between neighborhood disadvantage and readmission extends the knowledge base in this area and offers an opportunity to develop and test interventions targeting social determinants of health in the Medicare-enrolled CDI population. Most other studies of interventions designed to prevent readmissions have focused on acute myocardial infarction, pneumonia, and congestive heart failure [22, 39]. Infectious conditions (other than pneumonia) have not been included in these interventions. In the case of CDI, where symptoms may be prolonged or recurrent and the implications of readmissions extend beyond the individual patient, additional interventions like those used for chronic conditions may be useful.

To develop such interventions, further research is required to understand the precise mechanisms by which neighborhood disadvantage affects readmission risk in CDI patients. These mechanisms require additional research specifically designed to explore them since those proposed in this study remain conceptual. The actual mechanisms of increased risk, which could resemble those proposed earlier, likely differ somewhat from those affecting chronic conditions because of the infectiousness and recurrence patterns of CDI. Future research efforts should subsequently focus on developing and testing interventions to prevent readmissions of CDI patients living in disadvantaged neighborhoods such as specialized allocation of resources to improve the transition of care after hospitalization and access to follow-up in the outpatient setting. Studies should then examine the impact of these new interventions on CDI rates in healthcare institutions and how these interventions affect other healthcare-associated infections. This is especially important regarding other infections associated with high rates of recurrence, or with extensive or crucial post-discharge care procedures.

Our study has limitations. We did not have patient data on treatment factors, lab tests and vital signs to include in the analyses, any of which could explain the relationship between ADI and readmission risk. Given the lack of data on direct quality measures that may impact the risk of readmission, we could not analyze the quality of care as a marker for readmission in patients with a CDI-related index stay. Our choice of the 85th ADI national percentile as the threshold to split our cohort may have also impacted the findings from our study. However, other studies have used various methods for grouping their cohorts by ADI national percentile, often focusing on the 85th ADI national percentile and up [22, 40]. Another limitation of this study is the reliance on ICD-9 codes to indicate CDI, rather than lab data. However, ICD-9 codes have been shown to have reasonable sensitivity and specificity for indicating a diagnosis of CDI [41]. This dataset also does not allow us to identify planned readmissions. Planned readmissions (for non-CDI related purposes) could bias the effect size of any of our independent variables if planned readmissions are not uniformly distributed across our covariates; however planned readmissions in patients with CDI are not common. Focusing this study on Medicare patients may also be a limitation. However, as advanced age is considered a risk factor for CDI susceptibility, it is likely that the Medicare population well reflects the overall CDI susceptible population. Finally, similar to most other studies focusing on the Medicare population, we considered all cause readmissions and did not determine relatedness to CDI [18, 22]. A justification for this approach is that CDI may influence readmission even if it is not considered as the primary cause of it, as might occur in patients with partially resolved CDI at the time of discharge. Anorexia, dehydration, and weakness related to CDI may exacerbate other chronic comorbidities and lead to readmission. These limitations notwithstanding, this study is among the first to show that neighborhood disadvantage is associated with an increased risk of readmission in inpatients with an acute infectious transmissible condition such as CDI.

## Conclusions

Residence in a disadvantaged neighborhood significantly increases the risk of readmission in patients with an index CDI-related hospital stay. The effect size of neighborhood disadvantage was similar to those of chronic conditions and individual dual Medicare-Medicaid enrollment. Interventions that target the aggravating mechanisms of neighborhood disadvantage on CDI outcomes should be considered. Programs that are designed to reduce unwanted readmissions for chronic conditions, several of which are already in place in many healthcare institutions, may also benefit patients with CDI and should be evaluated for their impact on this population.

## Supplementary information

**Additional file 1: Supplementary Table 1.** Odds of 30-day readmission for CDI patients by patient characteristic and ADI score national percentile.

**Additional file 2: Supplementary Table 2.** Sensitivity analysis of risk of readmission by ADI grouping for varying threshold.

**Additional file 3: Supplementary Table 3.** Odds of 30-day rehospitalization by subgroup.

## Data Availability

The Medicare data that support the findings of this study are available from the Centers for Medicare & Medicaid Services but restrictions apply to the availability of these data, which were used under license for the current study, and so are not publicly available. The neighborhood disadvantage datasets analyzed during the current study are available in the University of Wisconsin Madison Neighborhood Atlas, https://www.neighborhoodatlas.medicine.wisc.edu/.

## Abbreviations

CDI: *Clostridioides difficile* infection
CMS: Centers for Medicare & Medicaid Services
ADI: Area deprivation index
SNF: Skilled nursing facility
