# Supplementary material for: Neighborhood disadvantage and 30-day readmission risk following Clostridioides difficile infection hospitalization
Source: BMC Infect Dis. 2020 Oct 16;20:762. doi: 10.1186/s12879-020-05481-x (PMC7565791; doi:10.1186/s12879-020-05481-x)
Supplement: Supplementary file 1 — Additional file 1: Supplementary Table 1. Odds of 30-day readmission for CDI patients by patient characteristic and ADI score national percentile. [file 12879_2020_5481_MOESM1_ESM.docx]

**Supplementary Table 1: Odds of 30-day readmission for CDI patients by patient characteristic and ADI score national percentile**

| **Variable** | **Odds ratio (95% CI)** | **Predicted Probability (%)**  **(95% CI)** |
| --- | --- | --- |
|  |  |  |
| Unadjusted | | |
| ADI < 85 percentile | Reference | 21.3 (20.7, 21.9) |
| ADI ≥ 85 percentile | 1.32 (1.20, 1.45) | 26.3 (24.5, 28.1) |
| Adjusted | | |
| **Patients** | | |
| Neighborhood disadvantage | | |
| ADI < 85 percentile | Reference | 21.6 (21.0, 22.2) |
| ADI ≥ 85 percentile | 1.16 (1.04, 1.28) | 24.1 (22.4, 25.8) |
| Age | | |
| 65+ y | Reference | 21.5, (20.8, 22.2) |
| 18-65 y | 1.14 (1.01, 1.29) | 23.8, (22.0, 25.6) |
| Sex | | |
| Male | Reference | 22.0, (21.0, 23.0) |
| Female | 0.99 (0.92, 1.07) | 21.9, (21.1, 22.6) |
| Race | | |
| White | Reference | 21.8, (21.1, 22.4) |
| Black | 1.08 (0.96, 1.20) | 23.0, (21.3, 24.7) |
| Other/Unknown Race | 0.98 (0.76, 1.26) | 21.6, (19.6, 23.6) |
| Medicaid Enrollment | | |
| No Medicaid in past 12 months | Reference | 21.2, (20.5, 21.9) |
| Medicaid in past 12 months | 1.15 (1.06, 1.25) | 23.6, (22.4, 24.8) |
| Disability | | |
| Not disabled | Reference | 21.6, (20.8, 22.4) |
| Disabled | 1.05 (1.16, 1.00) | 22.5, (21.2, 23.8) |
| Patient RUCA | | |
| Urban | Reference | 22.2, (21.5, 22.9) |
| Suburban | 0.87 (0.77, 0.99) | 20.0, (18.0, 21.9) |
| Large rural | 0.85 (0.67, 1.09) | 20.9, (19.1, 22.7) |
| Small rural | 1.11 (0.75, 1.65) | 22.8, (20.7, 24.9) |
| Elixhauser comorbidities | | |
| Hypertension | 1.06 (0.96, 1.17) | 22.1, (21.4, 22.7) |
| Fluid and electrolyte disorders | 1.12 (1.03, 1.22) | 22.6, (21.8, 23.4) |
| Deficiency anemia | 1.13 (1.04, 1.23) | 22.8, (22.0, 23.6) |
| Diabetes (without chronic complications) | 1.06 (0.97, 1.15) | 22.5, (21.5, 23.5) |
| Renal failure | 1.19 (1.10, 1.29) | 23.7, (22.6, 24.8) |
| Chronic pulmonary disease | 1.04 (0.96, 1.12) | 22.3, (21.3, 23.3) |
| Congestive heart failure | 1.04 (0.95, 1.13) | 22.3, (21.2, 23.4) |
| Depression | 1.02 (0.94, 1.11) | 22.2, (21.0, 23.3) |
| Other neurological conditions | 1.02 (0.94, 1.11) | 22.2, (21.0, 23.4) |
| Hypothyroidism | 0.99 (0.91, 1.08) | 21.8, (20.6, 23.0) |
| Peripheral vascular disease | 1.08 (0.99, 1.17) | 22.9, (21.6, 24.1) |
| Weight loss | 1.00 (0.92, 1.09) | 22.0, (20.7, 23.2) |
| Obesity | 1.03 (0.94, 1.13) | 22.3, (21.0, 23.7) |
| Diabetes (with chronic complications) | 1.12 (1.01, 1.25) | 23.5, (21.9, 25.1) |
| Valvular disease | 1.02 (0.93, 1.13) | 22.3, (20.7, 23. 8) |
| Metastatic cancer | 1.03 (0.86, 1.23) | 22.3, (19.4, 25.3) |
| Alcohol abuse | 1.13 (0.96, 1.33) | 23.9, (21.1, 26.7) |
| Drug abuse | 1.39 (1.18, 1.64) | 27.6, (24.5, 30.7) |
| Chronic blood loss anemia | 1.06 (0.90, 1.25) | 22.8, (20.1, 25.6) |
| Lymphoma | 1.22 (1.01, 1.48) | 25.3, (21.9, 28.8) |
| Acquired immune deficiency syndrome | 1.00 (0.68, 1.49) | 22.0, (15.4, 28.6) |
| Pulmonary circulation disease | 1.06 (0.94, 1.19) | 22.8, (20.9, 24.6) |
| Rheumatoid arthritis | 1.20 (1.06, 1.35) | 24.8, (22.6, 26.9) |
| Paralysis | 1.04 (0.91, 1.18) | 22.5, (20.4, 24.5) |
| Liver disease | 1.15 (1.00, 1.31) | 24.1, (21.8, 26.3) |
| Solid tumor without metastasis | 1.02 (0.91, 1.14) | 22.2, (20.4, 24.0) |
| Psychoses | 0.93 (0.83, 1.04) | 20.9, (19.2, 22.5) |
| Coagulopathy | 1.10 (1.00, 1.22) | 23.3, (21.8, 24.9) |
| **Index stay** | | |
| Length of stay | | |
| ≤ 2 days | Reference | 19.0, (17.0, 21.0) |
| 3-4 days | 1.13 (0.97, 1.32) | 20.9, (19.6, 22.2) |
| 5-6 days | 1.25 (1.07, 1.46) | 22.6, (21.2, 23.9) |
| 7+ days | 1.24 (1.08, 1.43) | 22.5, (21.7, 23.3) |
| SNF Discharge | | |
| Not discharged to SNF | Reference | 21.3, (20.6, 22.1) |
| Discharged to SNF | 1.10 (1.02, 1.18) | 22.9, (21.9, 23.9) |
| **Index Hospital** | | |
| Medical school affiliation | | |
| No medical school affiliation | Reference | 21.9, (21.0, 22.8) |
| Minor Medical School affiliated | 0.92 (0.84, 1.01) | 20.5, (19.4, 21.7) |
| Major Medical School affiliated | 1.07 (0.98, 1.17) | 23.1, (22.0, 24.2) |
| Hospital type | | |
| Non-profit/Government | Reference | 22.0, (21.4, 22.6) |
| For profit | 0.96 (0.86, 1.06) | 21.3, (19.6, 22.9) |
| Discharge volume | | |
| Discharge volume tertile 1 (highest) | Reference | 22.2, (21.5, 22.9) |
| Discharge volume tertile 2 (middle) | 0.95 (0.87, 1.04) | 21.3, (20.1, 22.6) |
| Discharge volume tertile 3 (lowest) | 0.83 (0.61, 1.15) | 20.7, (18.3, 23.1) |

ADI = Area Deprivation Index

RUCA = Rural -Urban Commuting Area

SNF = Skilled nursing facility
