# Supplementary material for: Neighborhood disadvantage and 30-day readmission risk following Clostridioides difficile infection hospitalization
Source: BMC Infect Dis. 2020 Oct 16;20:762. doi: 10.1186/s12879-020-05481-x (PMC7565791; doi:10.1186/s12879-020-05481-x)
Supplement: Supplementary file 2 — Additional file 2: Supplementary Table 2. Sensitivity analysis of risk of readmission by ADI grouping for varying threshold. [file 12879_2020_5481_MOESM2_ESM.docx]

**Supplementary Table 2: Sensitivity analysis of risk of readmission by ADI grouping for varying threshold**

| ADI Threshold | Adjusted Odds ratio (95% CI), Reference: patients below threshold |
| --- | --- |
| 95 | 1.07 (0.89, 1.27) |
| 85 | 1.16 (1.04, 1.28) |
| 75 | 1.14 (1.05, 1.25) |
| 65 | 1.14 (1.05, 1.23) |
| 55 | 1.10 (1.02, 1.19) |
| 50 | 1.11 (1.03, 1.19) |
| 45 | 1.08 (1.00, 1.16) |
| 35 | 1.05 (0.97, 1.14) |
| 25 | 1.04 (0.96, 1.14) |
| 15 | 1.05 (0.94, 1.17) |
| 5 | 1.01 (0.83, 1.23) |

ADI = Area Deprivation Index
