# Supplementary material for: Neighborhood disadvantage and 30-day readmission risk following Clostridioides difficile infection hospitalization
Source: BMC Infect Dis. 2020 Oct 16;20:762. doi: 10.1186/s12879-020-05481-x (PMC7565791; doi:10.1186/s12879-020-05481-x)
Supplement: Supplementary file 3 — Additional file 3: Supplementary Table 3. Odds of 30-day rehospitalization by subgroup. [file 12879_2020_5481_MOESM3_ESM.docx]

**Supplementary Table 3: Odds of 30-day rehospitalization by subgroup**

|  | Not discharged to SNF | Discharged to SNF | Not Medicare-Medicaid dual enrolled | Medicare-Medicaid dual enrolled | White | Black | Other/  Unknown |
| --- | --- | --- | --- | --- | --- | --- | --- |
|  | Adjusted OR (95% CI) | | Adjusted OR (95% CI) | | Adjusted OR (95% CI) | | |
| **ADI < 85 percentile** | Reference | Reference | Reference | Reference | Reference | Reference | Reference |
| **ADI ≥ 85 percentile** | 1.19 (1.05, 1.35) | 1.04 (0.88, 1.24) | 1.20 (1.04, 1.38) | 1.11 (0.95, 1.29) | 1.19 (1.04, 1.36) | 1.04 (0.84, 1.28) | 1.25 (0.93, 1.69) |
| **Ratio of Odds Ratios (95% CI)** | 1.14 (0.92, 1.41) | | 1.08 (0.88, 1.34) | | White & Black: 1.15 (0.90, 1.47) White & Other/Unknown: 0.95 (0.68, 1.31) | | |

ADI = Area Deprivation Index

SNF = Skilled nursing facility
